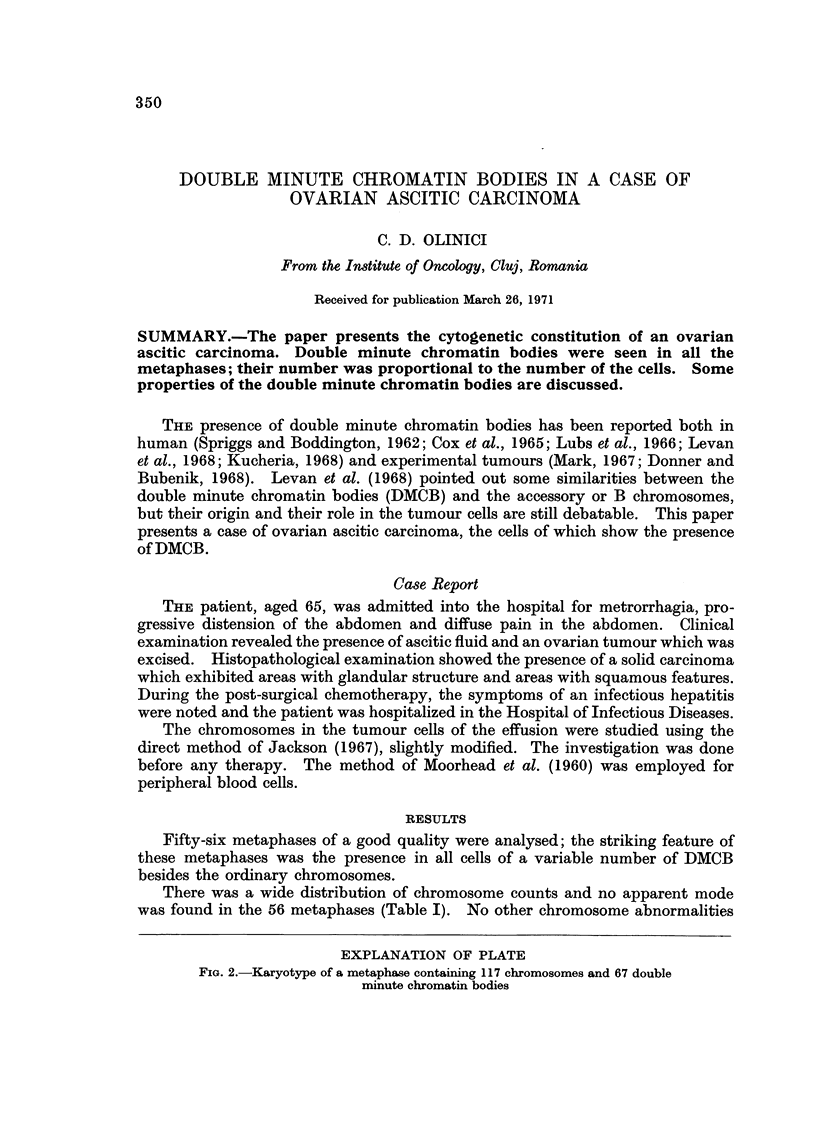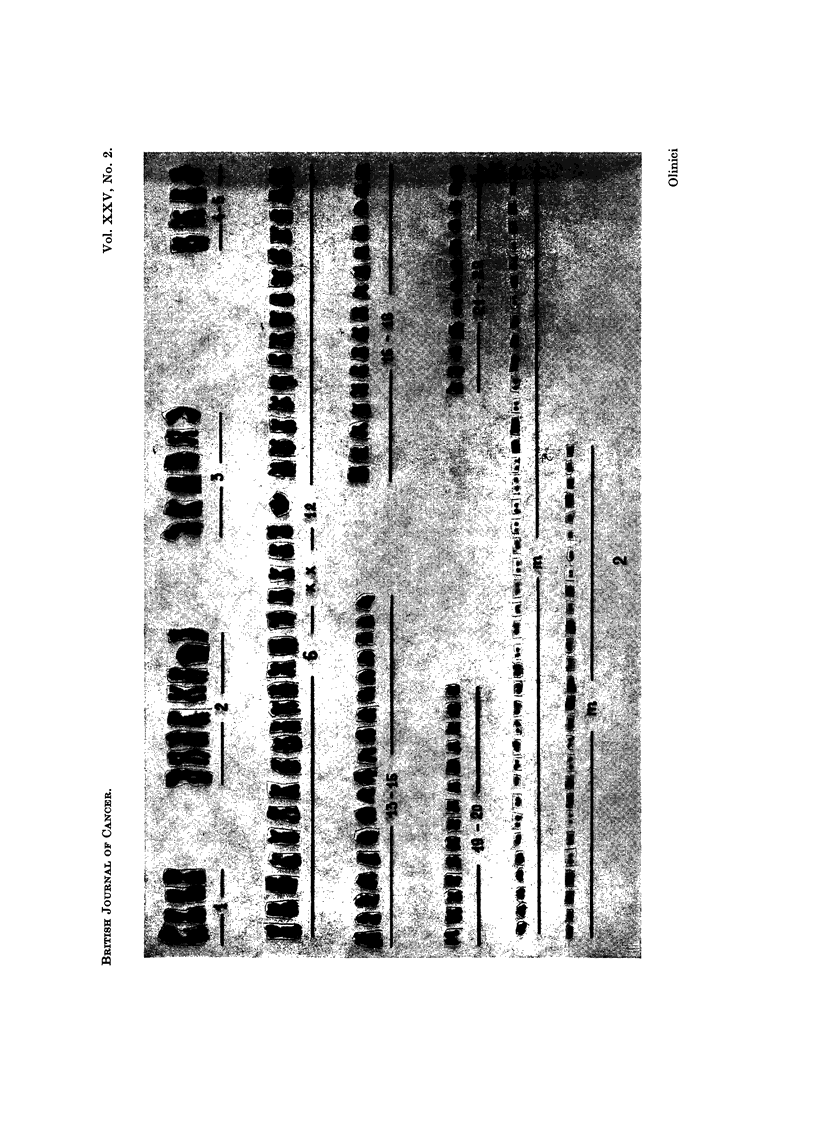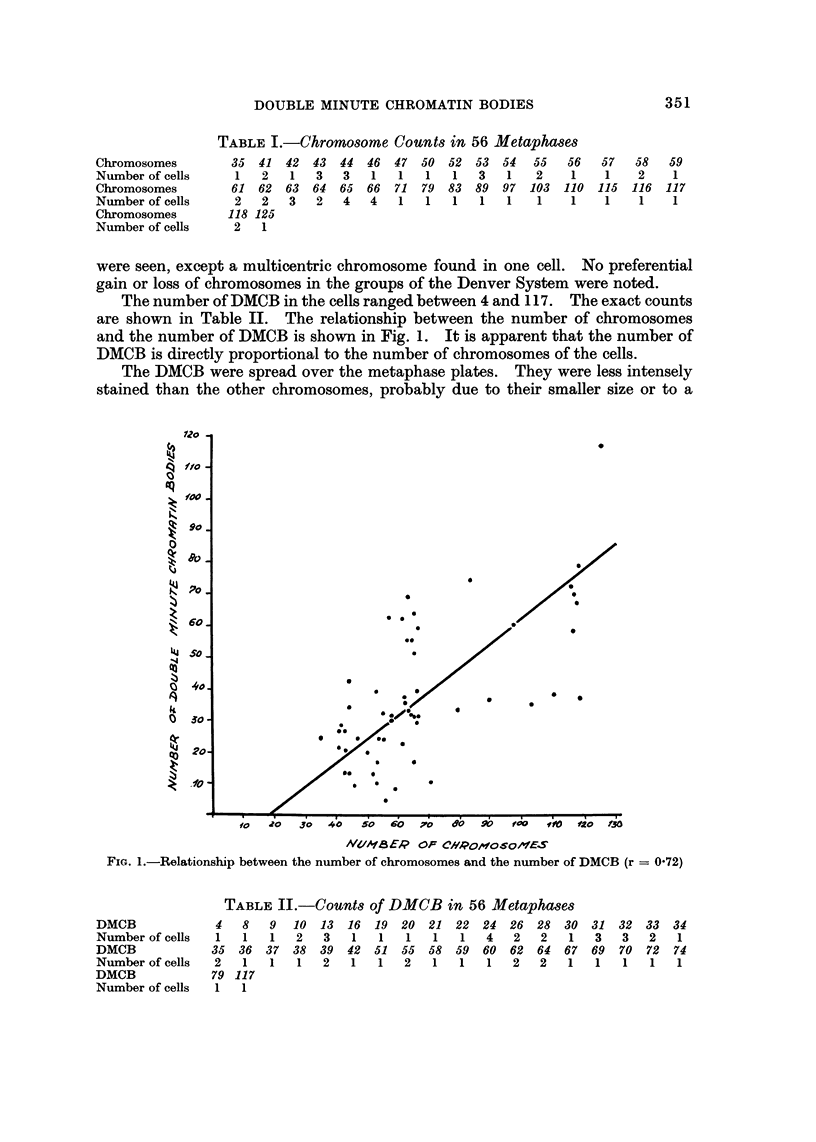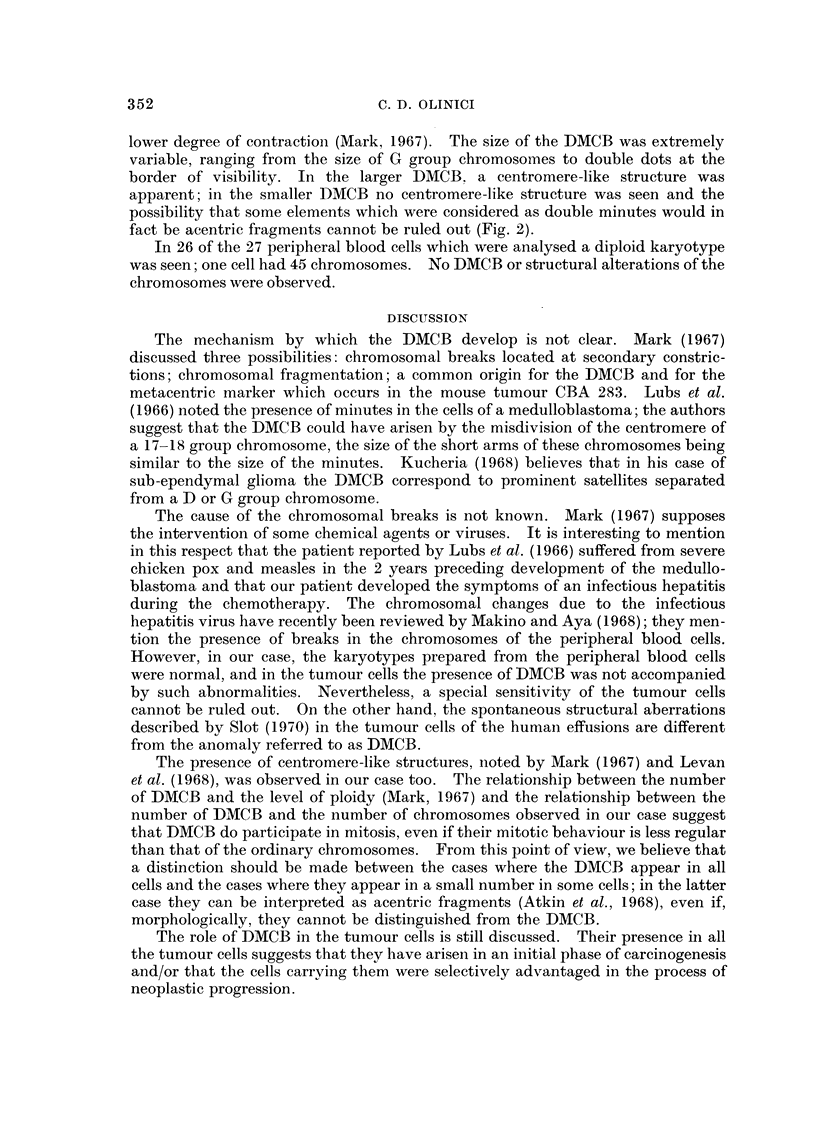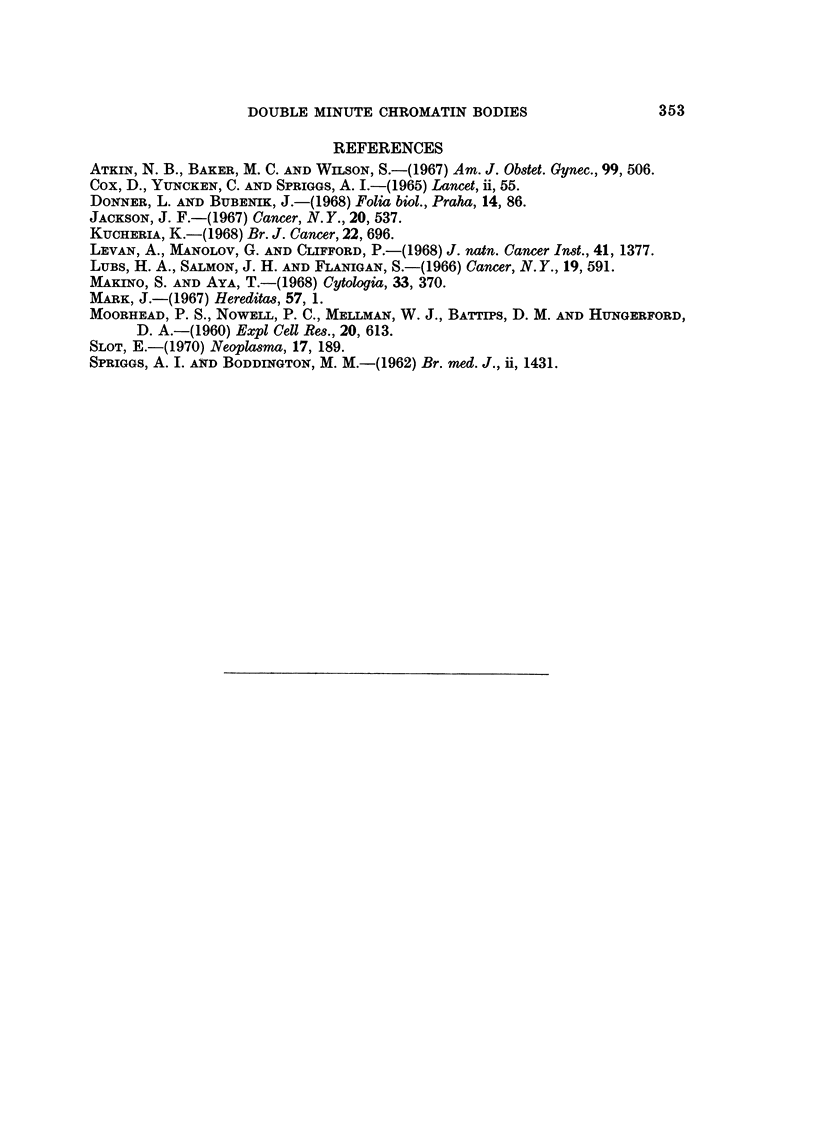# Double Minute Chromatin Bodies in a Case of Ovarian Ascitic Carcinoma

**DOI:** 10.1038/bjc.1971.45

**Published:** 1971-06

**Authors:** C. D. Olinici

## Abstract

**Images:**


### DOUBLE MINUTE CHROMATIN BODIES IN A CASE OF OVARIAN ASCITIC CARCINOMA

C. D. OLINICI

*From the Institute of Oncology, Cluj, Romania*

Received for publication March 26, 1971

**SUMMARY.—The paper presents the cytogenetic constitution of an ovarian ascitic carcinoma. Double minute chromatin bodies were seen in all the metaphases; their number was proportional to the number of the cells. Some properties of the double minute chromatin bodies are discussed.**

THE presence of double minute chromatin bodies has been reported both in human (Spriggs and Boddington, 1962; Cox *et al.*, 1965; Lubs *et al.*, 1966; Levan *et al.*, 1968; Kucheria, 1968) and experimental tumours (Mark, 1967; Donner and Bubenik, 1968). Levan *et al.* (1968) pointed out some similarities between the double minute chromatin bodies (DMCB) and the accessory or B chromosomes, but their origin and their role in the tumour cells are still debatable. This paper presents a case of ovarian ascitic carcinoma, the cells of which show the presence of DMCB.

### *Case Report*

THE patient, aged 65, was admitted into the hospital for metrorrhagia, progressive distension of the abdomen and diffuse pain in the abdomen. Clinical examination revealed the presence of ascitic fluid and an ovarian tumour which was excised. Histopathological examination showed the presence of a solid carcinoma which exhibited areas with glandular structure and areas with squamous features. During the post-surgical chemotherapy, the symptoms of an infectious hepatitis were noted and the patient was hospitalized in the Hospital of Infectious Diseases.

The chromosomes in the tumour cells of the effusion were studied using the direct method of Jackson (1967), slightly modified. The investigation was done before any therapy. The method of Moorhead *et al.* (1960) was employed for peripheral blood cells.

### RESULTS

Fifty-six metaphases of a good quality were analysed; the striking feature of these metaphases was the presence in all cells of a variable number of DMCB besides the ordinary chromosomes.

There was a wide distribution of chromosome counts and no apparent mode was found in the 56 metaphases (Table I). No other chromosome abnormalities

---

EXPLANATION OF PLATE

FIG. 2.—Karyotype of a metaphase containing 117 chromosomes and 67 double minute chromatin bodies

Olinici

TABLE I.—*Chromosome Counts in* 56 *Metaphases*

| | | | | | | | | | | | | | | | | |
|---|---|---|---|---|---|---|---|---|---|---|---|---|---|---|---|---|
| Chromosomes | *35* | *41* | *42* | *43* | *44* | *46* | *47* | *50* | *52* | *53* | *54* | *55* | *56* | *57* | *58* | *59* |
| Number of cells | 1 | 2 | 1 | 3 | 3 | 1 | 1 | 1 | 1 | 3 | 1 | 2 | 1 | 1 | 2 | 1 |
| Chromosomes | *61* | *62* | *63* | *64* | *65* | *66* | *71* | *79* | *83* | *89* | *97* | *103* | *110* | *115* | *116* | *117* |
| Number of cells | 2 | 2 | 3 | 2 | 4 | 4 | 1 | 1 | 1 | 1 | 1 | 1 | 1 | 1 | 1 | 1 |
| Chromosomes | *118* | *125* | | | | | | | | | | | | | | |
| Number of cells | 2 | 1 | | | | | | | | | | | | | | |

were seen, except a multicentric chromosome found in one cell. No preferential gain or loss of chromosomes in the groups of the Denver System were noted.

The number of DMCB in the cells ranged between 4 and 117. The exact counts are shown in Table II. The relationship between the number of chromosomes and the number of DMCB is shown in Fig. 1. It is apparent that the number of DMCB is directly proportional to the number of chromosomes of the cells.

The DMCB were spread over the metaphase plates. They were less intensely stained than the other chromosomes, probably due to their smaller size or to a

FIG. 1.—Relationship between the number of chromosomes and the number of DMCB (r = 0·72)

TABLE II.—*Counts of DMCB in* 56 *Metaphases*

| | | | | | | | | | | | | | | | | | | |
|---|---|---|---|---|---|---|---|---|---|---|---|---|---|---|---|---|---|---|
| DMCB | *4* | *8* | *9* | *10* | *13* | *16* | *19* | *20* | *21* | *22* | *24* | *26* | *28* | *30* | *31* | *32* | *33* | *34* |
| Number of cells | 1 | 1 | 1 | 2 | 3 | 1 | 1 | 1 | 1 | 1 | 4 | 2 | 2 | 1 | 3 | 3 | 2 | 1 |
| DMCB | *35* | *36* | *37* | *38* | *39* | *42* | *51* | *55* | *58* | *59* | *60* | *62* | *64* | *67* | *69* | *70* | *72* | *74* |
| Number of cells | 2 | 1 | 1 | 1 | 2 | 1 | 1 | 2 | 1 | 1 | 1 | 2 | 2 | 1 | 1 | 1 | 1 | 1 |
| DMCB | *79* | *117* | | | | | | | | | | | | | | | | |
| Number of cells | 1 | 1 | | | | | | | | | | | | | | | | |

lower degree of contraction (Mark, 1967). The size of the DMCB was extremely variable, ranging from the size of G group chromosomes to double dots at the border of visibility. In the larger DMCB, a centromere-like structure was apparent; in the smaller DMCB no centromere-like structure was seen and the possibility that some elements which were considered as double minutes would in fact be acentric fragments cannot be ruled out (Fig. 2).

In 26 of the 27 peripheral blood cells which were analysed a diploid karyotype was seen; one cell had 45 chromosomes. No DMCB or structural alterations of the chromosomes were observed.

### DISCUSSION

The mechanism by which the DMCB develop is not clear. Mark (1967) discussed three possibilities: chromosomal breaks located at secondary constrictions; chromosomal fragmentation; a common origin for the DMCB and for the metacentric marker which occurs in the mouse tumour CBA 283. Lubs *et al.* (1966) noted the presence of minutes in the cells of a medulloblastoma; the authors suggest that the DMCB could have arisen by the misdivision of the centromere of a 17–18 group chromosome, the size of the short arms of these chromosomes being similar to the size of the minutes. Kucheria (1968) believes that in his case of sub-ependymal glioma the DMCB correspond to prominent satellites separated from a D or G group chromosome.

The cause of the chromosomal breaks is not known. Mark (1967) supposes the intervention of some chemical agents or viruses. It is interesting to mention in this respect that the patient reported by Lubs *et al.* (1966) suffered from severe chicken pox and measles in the 2 years preceding development of the medulloblastoma and that our patient developed the symptoms of an infectious hepatitis during the chemotherapy. The chromosomal changes due to the infectious hepatitis virus have recently been reviewed by Makino and Aya (1968); they mention the presence of breaks in the chromosomes of the peripheral blood cells. However, in our case, the karyotypes prepared from the peripheral blood cells were normal, and in the tumour cells the presence of DMCB was not accompanied by such abnormalities. Nevertheless, a special sensitivity of the tumour cells cannot be ruled out. On the other hand, the spontaneous structural aberrations described by Slot (1970) in the tumour cells of the human effusions are different from the anomaly referred to as DMCB.

The presence of centromere-like structures, noted by Mark (1967) and Levan *et al.* (1968), was observed in our case too. The relationship between the number of DMCB and the level of ploidy (Mark, 1967) and the relationship between the number of DMCB and the number of chromosomes observed in our case suggest that DMCB do participate in mitosis, even if their mitotic behaviour is less regular than that of the ordinary chromosomes. From this point of view, we believe that a distinction should be made between the cases where the DMCB appear in all cells and the cases where they appear in a small number in some cells; in the latter case they can be interpreted as acentric fragments (Atkin *et al.*, 1968), even if, morphologically, they cannot be distinguished from the DMCB.

The role of DMCB in the tumour cells is still discussed. Their presence in all the tumour cells suggests that they have arisen in an initial phase of carcinogenesis and/or that the cells carrying them were selectively advantaged in the process of neoplastic progression.

### REFERENCES

Atkin, N. B., Baker, M. C. and Wilson, S.—(1967) *Am. J. Obstet. Gynec.*, **99**, 506.
Cox, D., Yuncken, C. and Spriggs, A. I.—(1965) *Lancet*, ii, 55.
Donner, L. and Bubenik, J.—(1968) *Folia biol., Praha*, **14**, 86.
Jackson, J. F.—(1967) *Cancer, N.Y.*, **20**, 537.
Kucheria, K.—(1968) *Br. J. Cancer*, **22**, 696.
Levan, A., Manolov, G. and Clifford, P.—(1968) *J. natn. Cancer Inst.*, **41**, 1377.
Lubs, H. A., Salmon, J. H. and Flanigan, S.—(1966) *Cancer, N.Y.*, **19**, 591.
Makino, S. and Aya, T.—(1968) *Cytologia*, **33**, 370.
Mark, J.—(1967) *Hereditas*, **57**, 1.
Moorhead, P. S., Nowell, P. C., Mellman, W. J., Battips, D. M. and Hungerford, D. A.—(1960) *Expl Cell Res.*, **20**, 613.
Slot, E.—(1970) *Neoplasma*, **17**, 189.
Spriggs, A. I. and Boddington, M. M.—(1962) *Br. med. J.*, ii, 1431.